# Responsiveness of European countries to the population mental health needs: A cross-national comparison study

**DOI:** 10.1192/j.eurpsy.2025.2448

**Published:** 2025-04-14

**Authors:** Celso Arango, Andrea Fiorillo, Geert Dom, Javier-David Lopez-Morinigo

**Affiliations:** 1 Department of Child and Adolescent Psychiatry, Institute of Psychiatry and Mental Health, Hospital General Universitario Gregorio Marañón, IiSGM, Centro de Investigación Biomédica en Red de Salud Mental (CIBERSAM), School of Medicine, Universidad Complutense, Madrid, Spain; 2 Department of Psychiatry, University of Campania “Luigi Vanvitelli”, Naples, Italy; 3 Collaborative Antwerp Psychiatric Research Institute (CAPRI), University of Antwerp, Antwerp, Belgium; 4 Department of Psychiatry, Hospital Universitario del Sureste, Arganda del Rey, Madrid, Spain

**Keywords:** Europe, healthcare disparity, mental health, policy evaluation, responsiveness

## Abstract

**Background:**

This study aimed to cross-compare European countries’ responsiveness to their populations’ mental health (MH) needs.

**Methods:**

For the EU-27 countries and the United Kingdom, the 2023 Headway Initiative collected data on 15 key performance indicators (KPIs) in responsiveness in healthcare, including workforce, facilities, quality of care, and MH expenditure, and 14 KPIs in responsiveness in workplaces, schools, and society. Bivariate correlations between Headway-transformed KPI scores, which were standardised in a 1–10 Likert Scale (1: worst performance; 10: best performance), tested for putative associations.

**Results:**

Responsiveness in healthcare: Sweden (10), Denmark (8.8), and Finland (8.3) showed the best performance, while Romania (1.0), Slovakia (1.1), and Latvia and Bulgaria (1.2) had the poorest performance. Responsiveness in workplaces: schools, and society, Germany (10.0), France (9.1), and Denmark (9.1) were the most responsive countries, while Greece and Slovakia (1.0) had the poorest responsiveness. MH status total scores negatively correlated with global scores on responsiveness in healthcare (*r* = −0.34, *p* = .075), workplaces (*r* = −0.46, *p* = .014), schools (*r* = −0.59, *p* = .003), and society (*r* = −0.53, *p* = .003) – poorer MH status, greater responsiveness.

**Conclusions:**

European countries significantly differed in their responsiveness to the populations’ MH needs, although the real effectiveness of their MH policies remains to be elucidated. Whether more responsive countries, which achieved poorer MH outcomes, successfully met greater preexisting MH needs, they failed to do so, or the relationship is driven by other third variables (e.g., quality of MH assessment) requires future investigation.

## Introduction

Despite the ongoing global mental health (MH) crisis, service provision continues to lag behind the need for care [1, 2]. The treatment gap represents a major public health issue [3, 4], which has widened over time [2]. In 2023, up to 25% of European citizens raised issues about mental healthcare, such as unacceptably long waiting lists, increased treatment costs and lack of information on service provision [5]. Of concern, investment in MH continues to lag behind other medical specialities, such as oncology and cardiology [6], and remains inequitably distributed within- and between-populations [2, 7]. It is true, however, that beyond a certain threshold, increased funding in mental healthcare may not achieve better outcomes, which may require greater social care expenditure [8]. In addition, stigma, among other unresolved issues, continues to prevent MH patients from receiving appropriate care [9–11].

In the first Headway-based article (Lopez-Morinigo et al., this issue), Europeans’ MH status was shown to significantly vary across countries, which was determined by a complex interplay of individual, environmental and social factors. A fundamental question therefore arises: *what can be done*? In short, two approaches can be adopted. First, universal primary prevention measures addressing the determinants of MH may reduce the incidence of mental disorders [7, 12], which was discussed in depth in the aforementioned article (Lopez-Morinigo et al., this issue). Second, from a secondary/tertiary prevention model, enhancing countries’ responsiveness to people’s MH needs should improve patient outcomes [13], which forms the context for this study. In brief, it can be anticipated that both increased MH funding and closer multiagency collaboration across social services, education, labour, and the justice system will be required [14].

In addition, our first Headway-based article in this issue (Lopez-Morinigo et al.) revealed that within the ongoing post-pandemic polycrisis [15], the impact of the determinants of MH differed between European countries, in line with previous reports [5, 16, 17]. Somehow surprisingly, the correlation between determinants of MH and the populations’ MH status was found to be weak across Europe, including “high-risk, good MH status” and “low-risk, poor MH status” countries (Lopez-Morinigo et al., this issue). Among other contributors, we speculated that between-country differences in their *responsiveness* to the population MH needs may partly explain this (Lopez-Morinigo et al., this issue). To test this hypothesis, we carried out the present cross-national comparison study of the EU-27 + UK countries’ responsiveness in healthcare, workplaces, schools, and society to their populations’ MH needs.

## Methods

### The 2023 Headway mental health index initiative

The Headway Initiative methodology was detailed elsewhere [15] (see also Lopez-Morinigo et al., this issue). Briefly, the 2023 Headway–Mental Health Index 3.0 collected data on 54 key performance indicators (KPIs) across the EU-27 + UK countries, including 14 KPIs in the responsiveness in healthcare and 15 KPIs in the responsiveness in workplaces, schools, and society. Data sources were official, authoritative, open-access datasets (e.g., World Health Organization [WHO]), and KPIs were decided by expert consensus meetings. Not only did this reduce the risk of potential selection bias, but also the use of open-access datasets ensured the replicability of the study, which is imperative in high-quality research.

For each country, KPI scores ranged from 1 (worst performance) to 10 (best performance) depending on the *relative* performance of each country compared with all other countries, thus making the data comparable across the board (see Lopez-Morinigo et al., this issue, for further details).

### Responsiveness in healthcare

Fourteen KPIs (see Table 1) were examined across four major domains: (i) MH workforce – rate of psychiatrists, child neuropsychiatrists, psychologists, and nurses per 100,000 inhabitants; (ii) mental healthcare facilities – rate of hospital beds, child and adolescent psychiatric beds, psychiatric hospitals, psychiatric units in general hospitals, and community-based MH facilities per 100,000 inhabitants; (iii) quality of care, which encompassed rate of hospital discharges from psychiatric wards, MH consultations and published psychiatric articles per 100,000 inhabitants, length of stay, and percentage of surveyed people reporting unmet needs; and (iv) economic resources for MH, measured as the percentage on total healthcare expenditure.

**Table 1.** KPIs in responsiveness to mental health needs in healthcare across European countries

Key performance indicators	Variable	Measure	Data source
*Workforce*	Psychiatrists	Rate per 100,000 inhabitants	Eurostat, 2021
	Child psychiatrists	Rate per 100,000 inhabitants	WHO mental health Atlas, 2020
	Psychologists	Rate per 100,000 inhabitants	WHO mental health Atlas, 2020
	Mental health nurses	Rate per 100,000 inhabitants	WHO mental health Atlas, 2020
*Facilities*	Psychiatric hospital beds	Rate per 100,000 inhabitants	Eurostat, 2021
	Child and adolescent inpatient beds	Rate per 100,000 inhabitants	Signorini et al. (2017)
	Psychiatric hospitals	Rate per 100,000 inhabitants	WHO, 2019
	Psychiatric units in general hospitals	Rate per 100,000 inhabitants	WHO, 2019
	Community mental health facilities	Rate per 100,000 inhabitants	WHO, 2019
*Quality of care*	Hospital discharge rates	Rate per 100,000 inhabitants	OECD
	Length of stay	Days	OECD
	Mental Health consultations	Rate per 100,000 inhabitants	Eurostat, 2019
	Unmet needs due to finances	%	Eurostat, 2019
	Published psychiatric articles	Rate per 100,000 inhabitants	Scimago, 2022
*Economic resources for mental health*	% of total health expenditure	% on Healthcare expenditure	WHO, 2011

URLs: Unreported URLs are due to data coming from the national ministry of health.

Eurostat, 2021: https://ec.europa.eu/eurostat/databrowser/view/hlth_rs_physcat__custom_11584649/default/table?lang=en

WHO Mental Health Atlas: https://apps.who.int/gho/data/view.main.HWF11v

Signorini et al., 2017: https://pubmed.ncbi.nlm.nih.gov/28596067/

WHO: https://apps.who.int/gho/data/node.main-eu.MHFAC?lang=en

OECD: data-explorer.oecd.org/vis?df[ds]=DisseminateFinalDMZ&df[id]=DSD_HEALTH_PROC%40DF_KEY_INDIC&df[ag]=OECD.ELS.HD&dq=.IMMUN…………….&pd=2010%2C&to[TIME_PERIOD]=false

Eurostat, 2019: https://ec.europa.eu/eurostat/databrowser/view/hlth_ehis_am6e/default/table?lang=en

Scimago, 2022: https://www.scimagojr.com/countryrank.php?year=2023&order=ci&ord=desc&category=2738

WHO, 2011: https://www.who.int/data/gho/data/indicators/indicator-details/GHO/government-expenditures-on-mental-health-as-a-percentage-of-total-government-expenditures-on-health-(---)

### Responsiveness in workplaces, schools, and society

Table 2 details the KPIs in responsiveness in workplaces, schools, and society, the variable(s) included in each KPI, the variable measure, and data source.

**Table 2.** Responsiveness to mental health needs in workplaces, schools, and society

Key performance indicators	Variable	Measure	Data source
Workplaces	Average gross wage for people with MD compared with those without MD	%	OECD, 2013
	Employment rate among people with MD	%	OECD, 2010
	Paid sick leave benefits for people with MD	Euros per inhabitant	Eurostat, 2021
	Unemployment benefits for people with MD	Euros per inhabitants	Eurostat, 2021*
	Job quality among people with MD	Survey score	Eurofound
	MH programs in workplaces	Number, type	WHO^1^
Schools	Community facilities for those with MD	Rate per 100,000 inhabitants	WHO^2^
	School dropouts among those with MD	%	OECD
	MH promotion programs	Number, type	WHO^3^
Society	Social workers	Rate per 100,000 inhabitants	WHO^4^
	Occupational therapists	Rate per 100,000 inhabitants	COTEC
	Social support	% of over–15 reporting poor social support	Eurostat, 2019
	Hospital beds	Rate per 100,000 inhabitants	Eurostat, 2022
	Disability benefits	Per capita expenditure for MD people	Eurostat
	MH promotion programs	Number, type	WHO

Abbreviation: MD, mental disorders.

URLs: Unreported URLs are due to data coming from the national ministry of health.

OECD, 2013: www.oecd-ilibrary.org/social-issues-migration-health/persons-with-mental-health-conditions-have-lower-wages-than-those-without_d8c41b45-en

OECD, 2010: www.oecd-ilibrary.org/sites/ea07586c-en/index.html?itemId=/content/component/ea07586c-en

Eurostat, 2021: https://ec.europa.eu/eurostat/databrowser/view/spr_exp_fsi__custom_11609858/default/table?lang=en

Eurostat, 2021*: ec.europa.eu/eurostat/databrowser/view/spr_exp_fsi__custom_11609858/default/table?lang=en

Eurofound: https://www.eurofound.europa.eu/en/data-catalogue/european-working-conditions-telephone-survey-2021-0

WHO1:

WHO2

OECD:

WHO3

WHO4: https://www.who.int/data/gho/data/indicators/indicator-details/GHO/social-workers-working-in-mental-health-sector-(per-100-000)

COTEC: https://www.coteceurope.eu/wp-content/uploads/2023/06/Summary-of-the-Profession-2023.pdf

Eurostat, 2019: https://ec.europa.eu/eurostat/databrowser/view/hlth_ehis_ss1b/default/table?lang=en&category=hlth.hlth_det.hlth_senv

Eurostat, 2022: https://ec.europa.eu/eurostat/databrowser/view/hlth_rs_bds1__custom_11597103/default/table?lang=en

Eurostat, 2021: https://ec.europa.eu/eurostat/databrowser/404-product/SPR_EXP_SUM__custom_256520?lang=en

In terms of responsiveness in *workplaces*, we looked at six KPIs, namely the wage gap between people with/without mental disorders, employment rate, sick leave benefits and unemployment benefits of people with mental disorders, job quality or satisfactions, and availability of MH promotion programs in workplaces.

Three KPIs in responsiveness to MH needs in *schools* were measured: availability of day centers for young people with mental disorders, percentage of youth dropping out of school due to MH issues, and availability of MH promotion programs in schools.

Regarding responsiveness in *society*, we evaluated six KPIs: availability of social workers and occupational therapists in the MH sector and hospital beds per 100,000 inhabitants, social support (measured as the proportion of people aged 15 years, who self-perceived poor social support), disability benefits for people with mental disorders, and existence of MH promotion programs for the general public.

### Statistics

Scores on all KPIs were reported per country, which were ordered alphabetically, including an overall EU-27 + UK average, at a descriptive level. We also ran a set of bivariate correlations between KPI scores to explore potential associations. Since all Headway-transformed KPI scores, which ranged from 1 to 10, followed a normal distribution, Pearson coefficients and the corresponding *p*-value were reported. Given the exploratory nature of the analyses, no correction for multiple testing techniques were applied to the correlations, which were unadjusted. The Statistical Package for Social Science, version 25.0 (SPSS Inc., Chicago, IL, USA), was used for all the above analyses. The significance level (two-tailed) was set at *p* < .05.

## Results

### Responsiveness in healthcare

Headway scores on KPIs in responsiveness in healthcare for the EU-27 + UK countries are detailed in Table 3.

**Table 3.** Headway initiative scores on key performance indicators in responsiveness in healthcare

	Workforce	Facilities	Quality of care	Economic resources	Final score
Austria	5.6	7.3	4.6	2.7	5.1
Belgium	5.4	3.4	4.8	2.1	3.1
Bulgaria	1.0	7.9	1.5	1.0	1.2
Croatia	3.3	8.0	3.0	2.7	3.7
Cyprus	3.0	9.6	7.5	3.1	6.4
Czechia	2.6	6.1	2.3	2.6	2.2
Denmark	8.3	6.7	10.0	3.6	8.8
Estonia	3.7	7.6	3.3	1.7	3.3
Finland	10.0	7.3	6.0	4.3	8.3
France	6.0	5.1	2.6	10.0	6.6
Germany	7.1	1.5	3.8	9.4	5.8
Greece	3.9	8.4	2.7	1.8	3.6
Hungary	2.5	5.9	2.5	1.9	1.8
Ireland	5.3	5.9	9.4	3.3	6.7
Italy	4.1	9.9	3.6	1.7	4.7
Latvia	2.7	4.1	1.5	3.1	1.2
Lithuania	5.6	7.7	2.4	2.7	4.3
Luxembourg	5.5	5.3	4.4	5.8	5.4
Malta	2.5	3.6	3.3	4.6	2.3
Netherlands	9.7	1.0	8.6	2.9	6.0
Poland	2.5	7.5	1.7	1.8	2.1
Portugal	1.9	8.7	4.0	3.5	4.2
Romania	2.0	6.2	1.0	1.8	1.0
Slovakia	2.1	5.5	1.4	2.2	1.1
Slovenia	2.8	10.0	2.4	3.9	4.6
Spain	2.5	7.3	3.2	3.3	3.4
Sweden	6.9	8.7	8.7	7.1	10.0
United Kingdom	4.1	7.5	7.1	5.2	6.7
EU–27 + UK	4.7	6.1	4.1	5.0	4.9

Overall, Sweden (10), Denmark (8.8), and Finland (8.3) had the best performance, while Romania (1.0), Slovakia (1.1), Latvia (1.2), and Bulgaria (1.2) had the poorest responsiveness.

In terms of overall workforce, Finland achieved the best performance (10.0), followed by the Netherlands (9.7) and Denmark, (8.3), while Bulgaria (1.0), Portugal (1.9), and Romania (2.0) had the poorest performance. Further details about the full raw data and Headway-transformed scores are provided in Table S1 in the online Supplementary Material.

With regard to facilities, Slovenia (10), Italy (9.9), and Cyprus (9.6) were the more resourced countries, whereas the Netherlands (1.0), Germany (1.5), and Belgium (3.4) had the poorest performance in facilities. The raw data and Headway-transformed scores are detailed in Table S2 (online Supplementary Material).

Regarding quality of care, Denmark (10.0), Ireland (9.4), and Sweden (8.7) showed the highest performance, whereas Romania (1.0), Slovakia (1.4), and Bulgaria and Latvia (1.5) had the poorest quality of care (see Table S3, online Supplementary Material, for further details).

In terms of MH expenditure (see also Table S4, online Supplementary Material), France, Germany, and Sweden spent 13.9, 13.1, and 10.0% of the total health expenditure on MH, respectively. On the other hand, Bulgaria (2%), Estonia (2.9%), and Italy (3.0%) showed the lowest percentage of MH expenditure.

### Responsiveness in workplaces, schools, and society

Headway scores on KPIs for responsiveness in workplaces, schools, and society are provided in Table 4.

**Table 4.** Headway-transformed KPI scores in responsiveness in workplaces, society, and schools

	Workplaces	Schools	Society	Final score
Austria	7.8	7.6	7.4	9.0
Belgium	9.4	4.0	6.1	6.4
Bulgaria	3.7	5.2	4.5	6.1
Croatia	5.4	6.7	3.7	4.7
Cyprus	5.6	2.2	3.7	3.5
Czechia	3.4	4.1	7.5	5.6
Denmark	8.9	7.5	7.2	9.1
Estonia	4.9	7.4	2.0	5.5
Finland	9.4	7.5	4.7	7.6
France	8.9	10.0	9.3	9.1
Germany	10.0	8.1	10.0	10.0
Greece	1.0	2.4	1.0	1.0
Hungary	3.4	9.0	7.5	8.0
Ireland	8.2	6.3	6.6	7.9
Italy	6.2	6.8	3.9	5.6
Latvia	4.5	8.6	4.8	7.3
Lithuania	4.8	8.6	7.2	7.5
Luxembourg	9.2	3.7	6.7	6.0
Malta	6.0	4.2	4.0	5.6
Netherlands	9.2	4.9	3.3	7.1
Poland	6.6	5.3	6.0	5.0
Portugal	5.0	7.3	6.0	7.5
Romania	4.0	6.0	3.1	6.3
Slovakia	2.4	1.0	3.5	1.0
Slovenia	4,3	7.9	5.7	7.0
Spain	4,7	5.1	4.5	4.1
Sweden	6,4	5.1	8.6	8.0
United Kingdom	4,5	8.4	6.2	7.6
EU–27 + UK	6,7	7.0	6.5	7.1

According to the total scores, Germany (10), France (9.1), and Denmark (9.1) achieved the best performance, while Slovakia (1.0), Greece (1.1), and Cyprus (3.5) had the poorest response.

Regarding KPIs in responsiveness in *workplaces*, Germany (10), Finland, and Belgium (9.4) reached the best performance, while Greece (1.0), Slovakia (1.4), and Czechia and Hungary (3.4) had the poorest response (see also Table S5, online Supplementary Material).

In terms of responsiveness in *schools*, France (10.0) and Latvia and Lithuania (8.6) had the best performance, whereas Slovakia (1.0), Cyprus (2.2), and Greece (2.4) had the poorest (see also Table S6, online Supplementary Material).

Regarding KPI scores in responsiveness in *society*, Germany (10), France (9.3), and Sweden (8.6) achieved the highest performance, while Greece (1.0), Estonia (2.0), and Romania (3.1) had the poorest performance (see Table S7 in the online Supplementary Material for further details).

Overall, for KPI scores in responsiveness in non-healthcare, Germany (10), France, and Denmark (9.1) were the most responsive countries, while Slovakia (1.0), Greece (1.0), and Cyprus (3.6) had the lowest scores.

## Relationship between MH status and responsiveness of the system

Bivariate correlations between status and determinant KPIs across EU-27 + UK countries are detailed in Table 5. While overall performance on healthcare responsiveness (*r* = −0.34, *p* = .075) did not reach statistical significance (although at a borderline level), responsiveness in workplaces (*r* = −0.46, *p* = .014), schools (*r* = −0.59, *p* < .001), and society (*r* = −0.53, *p* = .003) correlated with the overall status – the better the responsiveness, the worse the MH status. Further significant correlations emerged from the analyses (Table 5).

**Table 5.** Relationship between mental health status and responsiveness across EU-27 + UK countries

	Prevalence		Incidence		YLD_AD		YLD_20		Mortality		Suicide		Status	
	r	p	r	p	r	p	r	p	r	p	r	p	r	p
***Healthcare***	**−0.41**	**.030**	**−0.67**	**<.001**	−0.19	.323	−0.18	.367	**−0.43**	**.021**	0.01	.981	−0.34	.075
*Workforce*	−0.27	.170	**−0.48**	**.010**	−0.07	.724	−0.11	.586	**−0.38**	**.044**	−0.19	.329	**−0.49**	**.008**
Psychiatrists	−0.32	.093	**−0.39**	**.041**	−0.18	.366	−0.15	.433	−0.24	.214	−0.21	.272	−0.33	.089
Child psychiatrists	−0.19	.321	−0.32	.101	0.02	.927	−0.03	.897	−0.19	.330	−0.18	.370	−0.31	.108
Psychologists	−0.28	.144	−0.35	.069	−0.08	.675	−0.16	.400	**−0.40**	**.033**	−0.07	.734	−0.34	.075
Nurses	−0.01	.941	**−0.42**	**.025**	0.03	.864	0.03	.882	−0.33	.091	−0.14	.476	**−0.52**	**.005**
*Facilities*	−0.08	.681	0.10	.594	0.01	.970	0.07	.771	0.32	.096	0.07	.715	0.31	.106
Psych beds	−0.34	.072	−0.29	.131	−0.18	.346	−0.21	.292	0.01	.978	0.37	.052	0.30	.126
Child adoles beds	−0.23	.235	−0.12	.530	0.16	.411	−0.09	.665	0.15	.452	0.34	.073	0.39	.037
Psych Hospitals	−0.05	.579	0.24	.214	0.01	.980	−0.02	.924	−0.02	.922	0.14	.463	0.17	.373
Psych units	0.14	.550	−0.12	.590	−0.22	.338	−0.08	.724	0.06	.786	0.20	.395	0.29	.196
CMHTs	0.31	.107	**0.43**	**.021**	**−0.49**	**.008**	**0.49**	**.008**	**0.52**	**.017**	**−0.70**	**.001**	−0.31	.108
*Quality of care*	−0.31	.112	**−0.51**	**.005**	−0.12	.426	0.06	.744	−0.20	.296	0.15	.438	−0.17	.394
Hospital discharge	**−0.45**	**.015**	**−0.41**	**.031**	−0.45	.016	−0.25	.205	−0.09	.656	**0.46**	**.014**	0.26	.176
LOS	0.03	.860	0.12	.539	−0.09	.660	0.22	.252	0.13	.511	−0.10	.616	−0.04	.855
Consultations	−0.12	.535	−0.34	.079	−0.09	.653	0.16	.414	−0.28	.153	−0.18	.367	−0.36	.062
Unmet needs	−0.19	.330	−0.26	.185	−0.23	.249	−0.13	.508	−0.28	.160	0.25	.200	0.09	.625
Articles	**−0.41**	**.028**	**−0.73**	**<.001**	−0.23	.240	−0.16	.416	**−0.49**	**.008**	0.16	.426	−0.33	.083
*Health expenditure*	−0.16	.410	**−0.40**	**.032**	−0.13	.429	−0.19	.333	**−0.43**	**.024**	−0.05	.818	−0.27	.177
***Workplaces***	−0.19	.329	**−0.47**	**.012**	0.02	.916	−0.06	.761	**−0.49**	**.009**	−0.12	.541	**−0.46**	**.014**
Gross wage	0.27	.156	0.15	.459	0.34	.073	0.20	.304	0.01	.946	−0.10	.620	−0.13	.521
Employment rate	0.19	.333	0.02	.902	0.15	.443	0.11	.584	−0.13	.497	−0.12	.543	−0.17	.384
Sick leave benefits	−0.23	.229	−0.36	.058	−0.05	.787	−0.16	.415	**−0.41**	**.028**	−0.03	.873	−0.34	.073
Unemployment benefits	−0.33	.085	**−0.64**	**<.001**	−0.23	.236	−0.18	.362	−0.11	.581	0.11	.580	−0.01	.979
Promotion programs	−0.18	.361	−0.19	.334	0.02	.933	0.03	.866	−0.29	.129	−0.24	.225	**−0.40**	**.034**
Job quality	−0.16	.423	−0.32	.395	−0.07	.739	−0.17	.395	**−0.56**	**.002**	0.04	.847	−0.35	.067
***Schools***	0.03	.890	0.07	.726	0.30	.119	0.13	.516	−0.22	.257	**−0.63**	**.001**	**−0.59**	**.001**
Community facilities	−0.30	.115	**−0.37**	**.049**	−0.14	.485	−0.18	.365	−0.24	.218	−0.08	.684	−0.36	.061
School dropouts	0.18	.340	0.35	.064	0.44	.019	0.29	.129	−0.03	.878	**−0.43**	**.023**	−0.21	.294
Promotion programs	0.06	.742	0.04	.832	0.19	.336	0.06	.744	−0.17	.385	**−0.57**	**.001**	**−0.53**	**.003**
***Society***	0.08	.699	−0.14	.478	0.07	.717	−0.14	.462	−0.29	.133	**−0.39**	**.043**	**−0.53**	**.003**
Social workers	−0.28	.154	**−0.45**	**.017**	−0.14	.484	−0.13	.515	−0.22	.259	−0.10	.622	−0.25	.197
Occupational therapists	−0.02	.903	**−0.40**	**.037**	0.02	.937	0.01	.950	**−0.42**	**.026**	−0.05	.795	−0.41	.030
Social support	−0.21	.273	−0.35	.070	−0.15	.460	−0.18	.349	−0.11	.572	0.13	.449	−0.06	.766
Residential beds	**0.62**	**<.001**	**0.60**	**<.001**	0.34	.075	0.12	.529	0.31	.103	−0.26	.188	0.02	.929
Disability benefits	−0.14	.477	−0.35	.067	0.00	.99	−0.12	.529	**−0.38**	**.046**	−0.03	.882	−0.35	.067
Promotion programs	0.02	.913	−0.03	.889	0.02	.919	−0.03	.872	−0.26	.181	**−0.47**	**.011**	**−0.48**	**.010**
**Non-HC system**	−0.02	.912	−0.18	.367	0.16	.429	−0.08	.672	**−0.50**	**006**	**−0.48**	**.010**	**−0.71**	**<.001**

In bold, statistically significant (p < .05) correlations.

### Overall results

**Figure 1.**
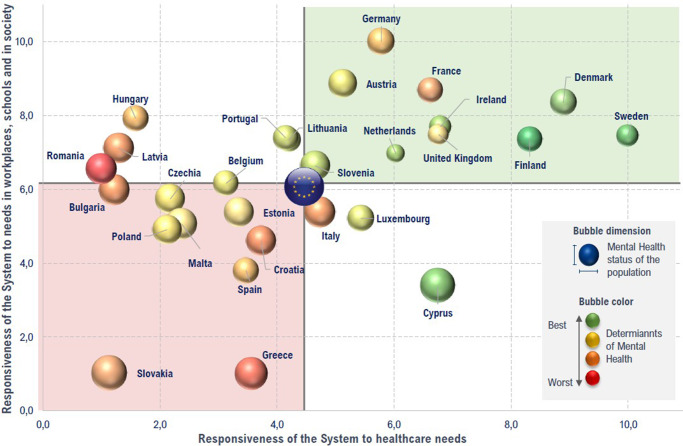
Overall results from the 2023 Headway Mental Health Index 3.0.

The overall results, including all KPIs in MH status, determinants, and responsiveness to MH needs, are graphically summarized in Figure 1. In particular, those countries in the top-right corner (in green color) achieved the best overall performance in MH-related KPIs, namely Sweden, Finland, and Denmark. Conversely, countries in the bottom-left quadrant (in red color) had the poorest overall performance in MH-related KPIs, such as Greece and Slovakia, although both countries had very good MH status (see bubble dimension, although it is noteworthy that the larger the bubble, the better the MH status), as discussed further below.

## Discussion

### Principal findings

This second Headway-based article revealed a high variation in the responsiveness to MH needs across EU-27 + UK countries, which was shown to negatively correlate with the population MH status – the poorer the MH status, the better the responsiveness. Thus, Sweden, Finland, Denmark, France, and Germany achieved the best performance on responsiveness to their populations’ MH needs, although their performance in MH status was poor. On the other hand, Greece, Slovakia, and Cyprus, which performed well in MH status KPIs, showed the poorest performance in responsiveness to MH needs. At first glance, it seems that countries’ responsiveness failed to mitigate the impact of the determinants of MH. Conversely, one may argue that better-performing countries in responsiveness might do so because of greater preexisting MH needs (e.g., Scandinavian countries) and vice versa (South-European countries). Nevertheless, tackling inequality and bridging the mental healthcare gap should guide future European MH policies.

### Responsiveness in healthcare

The WHO defined health systems *responsiveness* as “*how well the health system meets the legitimate expectations of population for non-health enhancing aspects of the health system*” [18]; hence, an inherent goal of any health system which must be measured and monitored over time [19]. More specifically, health system responsiveness encompasses both the system’s ability and capacity to respond and its actual response to medical [20] and nonmedical issues [18].

In particular, Scandinavian countries, such as Sweden (10), Denmark (8.8), and Finland (8.3), showed the best performance in responsiveness in healthcare, while Romania (1.0), Slovakia (1.1), and Latvia and Bulgaria (1.2) had the poorest results. Specifically, four major domains of healthcare systems across Europe were evaluated, namely workforce, facilities, quality of care, and economic resources.

Regarding workforce, Finland (10), the Netherlands (9.7), and Denmark (8.3) achieved the best performance, while Bulgaria (1.0), Portugal (1.9), and Romania (2.0) showed the lowest scores. As of 2021, on average, for the EU-27 + UK countries, there were 18.8 psychiatrists per 100,000 inhabitants, ranging from 10.2 (Bulgaria) to 28.6 (Germany), that is, an almost threefold variation. Two countries – Spain and Malta – had no child psychiatrists at all. Such a high variability of healthcare professionals’ rates across European countries may have widened since the coronavirus disease 2019 (COVID-19) pandemic [2–4]. Of concern, there has been a severe shortage of MH professionals as the number of individuals in need increased, which seems to be exacerbated by high levels of staff burnout [21]. Thus, it is estimated that by 2030, Europe will face a shortfall of ~600,000 doctors [22]. Although Digital Mental Health approaches may mitigate this [23–25], their long-term outcomes remain unknown [26]. Therefore, urgent action is needed to enhance staff recruitment and retention rates across Europe [22].

In terms of facilities, Slovenia (10), Italy (9.9), and Cyprus (9.6) were the most resourced countries, whereas the Netherlands (1.0), Germany (1.5), and Belgium (3.4) had the poorest performance. Given the Headway Initiative methodology [15], results of worse-performing countries may only indicate a better provision of inpatient, rather than community-based MH facilities, and not necessarily limited MH facilities.

In keeping with the above, mental healthcare expenditure significantly varied across Europe, with France (13.9%), Germany (13.1%), and Sweden (10.0%) – all of them with good performance in responsiveness – emerging as the principal investors, significantly exceeding the EU-27 + UK average of 5.4%. Hence, many European countries failed to comply with the 2018 Lancet Commission recommendations, according to which national MH budgets should receive between 5% (low- and middle-income countries) and 10% (high-income countries) of the total healthcare [27]. Certainly, greater national MH expenditure was linked to better quality of care in psychiatry [28], including lower suicide rates [29]. In addition, MH prevention yields a high long-term return on investment [30].

Regarding quality of care, Denmark (10.0), Ireland (9.4), and Sweden (8.7) showed the highest performance, whereas Romania (1.0), Slovakia (1.4), and Bulgaria and Latvia (1.5) had the poorest quality of care. Despite MH becoming a top priority in the political agenda of European governments and institutions [31], up to 6.2% Europeans reported unmet MH needs due to financial issues in 2022, ranging from 1.1% (Romania) to 29.8% (Finland). [5]. These *apparent* between-country differences, however, may well reflect the quality of data and reporting issues, including the influence of stigma, which tends to discourage people from talking openly about MH [11].

### Responsiveness in workplaces, schools, and society

Regarding responsiveness in workplaces, Germany (10) and Finland and Belgium (9.4) reached the best performance, while Greece (1.0), Slovakia (1.4), and Czechia and Hungary (3.4) had the lowest scores. Overall, almost two in three Europeans with mental disorders were found to be unemployed and get paid, on average, 30% lower wages than their counterparts. Indeed, MH patients face exclusion from work, largely due to stigma and discrimination, which prevents them from recovery [11, 32]. Labour market integration policies should be promoted [33, 34], which also reduced suicide risk [35], while community-based mental healthcare effects on return-to-work outcomes are less clear [36]. Although the WHO developed specific guidelines on MH promotion at work [37], guidelines compliance and their effects on health and social outcomes remain to be established [38].

In terms of responsiveness in schools, France (10.0) and Latvia and Lithuania (8.6) achieved the best performance, whereas Slovakia (1.0), Cyprus (2.2) and Greece (2.4) had the poorest results. It is well-established that for almost two in three MH patients, the illness onset occurs before age 25 years, with a peak at 14.5 years [39]. In Europe, full-time compulsory education/training usually lasts 10–11 years and ends at the age of 15–16 years [40]. Our results revealed that the overall performance on responsiveness in schools negatively correlated with the overall MH status and suicide rates – the better the performance at schools, the poorer the MH status, including higher suicide rates. However, up to 20% of European children were shown to experience MH problems during their school years, and a comparable proportion reported feelings of unhappiness and anxiety about the future, which was linked to bullying, challenging schoolwork and loneliness [5]. In addition, EU Countries widely differed in the ability to provide MH prevention and promotion programs at schools, and between 7.5% (Estonia) and up to 21.5% (The Netherlands) of school dropouts were children with mental disorders [41]. Hence, few would question the appropriateness of schools as “the right place at the right time” for early intervention [12, 42]. Consistent with this, targeted prevention programs reduced depression and anxiety symptoms [43], whereas universal school-based interventions prevented suicidal behaviour in adolescents [44]. In addition, school-based MH promotion programs focused on resilience may aid students in managing their own stress [45], which could be delivered through digital technologies [46]. MH clinical liaison teams appear to facilitate access to care [47, 48], which should result in better long-term outcomes, although this remains to be demonstrated.

Regarding responsiveness in society, Germany (10), France (9.3), and Sweden (8.6) achieved the highest performance, while Greece (1.0), Estonia (2.0), and Romania (3.1) had the poorest performance. Of note, there was a negative association between responsiveness in society, especially MH promotion programs, and MH status – the poorer the MH status, the better the responsiveness. From a societal perspective, stigma and discrimination were identified as the main areas of concern for improving people’s MH [11]. Stigma was defined as the co-occurrence of stereotyping, separating, status loss, and discrimination in the context of power inequities [49]. While the youth may particularly benefit from antistigma campaigns [50], such as the England-based Time-to-Change [51], especially in the short-term [51], their long-term effects remain far from clear [52].

### Next steps

In the first Headway-based article (Lopez-Morinigo et al., this issue), we examined the MH status of Europeans and its determinants. Interestingly, overall KPI scores on responsiveness in workplaces (*r* = −0.46, *p* = .014), schools (*r* = −0.59, *p* < .001), and society (*r* = −0.53, *p* = .003) negatively correlated with overall KPI scores on MH status – the better the responsiveness, the worse the MH status. On the other hand, the correlation between overall performance in responsiveness in healthcare and MH status did not reach significance (*r* = −0.34, *p* = .075). Taken together, these findings deserve some consideration. First, it seems that responsiveness to people’s MH needs may largely occur in workplaces, schools, and society rather than in healthcare systems. Second, there was a negative correlation between MH status and responsiveness – the better the status, the worse the responsiveness. Whether more responsive countries succeeded in meeting preexisting greater MH needs (i.e., a positive result) or they failed to address people’s MH issues (i.e., a negative result) remains to be clarified. Of relevance, overall KPI scores on determinants, which were not linked with MH status KPIs (Lopez-Morinigo et al., this issue), positively correlated with responsiveness in healthcare (*r* = 0.41, *p* = .031) and in workplaces (*r* = 0.40, *p* = .034). This noted, further theoretical debate about the appropriateness of KPIs for the evaluation of public MH policies is still warranted.

Indeed, the lack of comprehensive, independent, and comparable data poses a major barrier to the development of a monitoring and evaluation/accountability framework in MH policymaking. To address this challenge, within the United Nations Sustainable Development Goals agenda, the Countdown Global Mental Health 2030 was designed, which collected data on 48 indicators from 15 sources covering 193 countries across the globe. Specifically, indicators were clustered around three themes: determinants of MH and factors shaping MH needs and their response [53]. Easily-accessible datasets, such as the European Headway Initiative [15] and the Countdown Global Mental Health [53], may inform public MH policymaking, including between-country comparisons, whereas researchers may generate novel real-world hypotheses. However, these data-driven approaches are not exempt from criticism, namely the extent to which somehow arbitrarily chosen KPIs really align with the population MH needs and the time required to evaluate MH policy changes.

In particular, this study’s results may contribute to future evidence-based MH policies aimed to address European-level and country-specific challenges. To this end, scientific societies, such as the European Psychiatric Association (EPA) and the World Psychiatric Association (WPA), are committed to providing a strong leadership. This being said, it is worth noting that more than nine in 10 healthcare interventions subject to Cochrane Reviews were not supported by high-quality evidence, while harms do not tend to get published [54]. In addition, health authorities must bridge the gap between research evidence, clinical guideline recommendations, and approved interventions by regulatory bodies, especially in children and adolescents with serious mental illness [55, 56].

Of relevance, MH policymaking is not exempt from compliance with international codes of ethics in psychiatry [57]. To this end, the ongoing dialogue between the EPA and the WPA [58] and closer collaboration between national psychiatric associations [59] will be essential. Digital MH raises ethical issues about privacy and the use of personal data, which is particularly relevant to children and adolescents [60]. More broadly speaking, public MH also faces the challenge of appropriately timing and targeting interventions, which is crucial for staging models [61], not to mention the sometimes blurred boundaries between health and disease in MH [60].

To address the increased MH needs after the COVID-19 pandemic, some MH action plans have been made by European governments. For instance, since 2021, the UK government has offered training grants for senior MH leads to all state schools and colleges, whereas in Spain, a 24-h suicide prevention hotline became available that same year. In 2022, MH services in Belgium were reformed to facilitate access to care from schools, sports facilities, and work. One year later, the 2023 German Centre for Mental Health (DZPG) research network was launched. In Italy, since 2023/2024 a psychologist can be accessed both in schools and in primary care (i.e., “Psicologo di base”) [15].

In light of our results, further evidence-based public MH interventions can be recommended. To enhance responsiveness in healthcare systems, not only is higher MH expenditure required, but also optimization of available resources, such as the so-called digital MH [62]. Labour market integration policies may make the onset of work-related mental disorders less likely [33, 34]. In schools, MH promotion programs [46] and clinical liaison teams [48] may become the key primary and secondary (i.e., early detection/intervention) preventive strategies, respectively. Finally, from a societal approach, antistigma campaigns should facilitate professional help-seeking by MH patients [11]. See Table 6 for further details.

**Table 6.** Interventions on KPIs in responsiveness to mental health needs in healthcare, workplaces, schools, and society

	Interventions	Type of prevention	Level of evidence	References
*Healthcare*	Digital mental health	Secondary and tertiary	Umbrella review	[62]
	Increased expenditure on MH	Secondary and tertiary	National-based studies	[29]; [28]
*Workplaces*	Labor market integration policies	Universal primary	National-based studies	[33]; [34]
*Schools*	Clinical liaison teams	Secondary and selected/indicated	Systematic review	[48]
	MH promotion programs	Universal primary	Meta-analysis	[46]
Society	Antistigma campaigns	Universal primary	Further research is needed	[11]

### Strengths and limitations

The Headway Initiative collected cross-national population-level data on 54 MH-related KPIs, including MH status and determinants (Lopez-Morinigo et al., this issue) and responsiveness of the systems to the population MH needs. Data came from official, authoritative open-access datasets, such as Eurostat, WHO, or the Organisation for Economic Co-operation and Development (OECD), thus ensuring the replicability of the study. Owing to this methodology, some evidence-based public MH interventions were recommended.

However, three main limitations of this study should be acknowledged. First, the Headway methodology relied on national datasets, which differed in the quality of data and data availability (i.e., under- and overreporting issues), including cross-national cultural differences. Further, data on KPIs were collected in different years depending on data availability, that is, from the most recent year when the variable data were available. In addition, it is worth noting that “association does not mean causation” [63], and caution is needed when inferring causality from the above bivariate correlations. Second, further nontested KPIs, such as psychiatric medication availability and compliance and/or funding source of healthcare systems, may have altered the results. Third, the Headway Initiative adopted both analytical and qualitative approaches, which, although unlikely, may have biased the results. In particular, the Headway Mental Health Index aimed to comparatively measure European countries’ relative performance on a number of MH-related KPIs, thus ranking them rather than cross-comparing raw data on the above variables.

## Conclusions

The world is facing an unprecedented MH crisis, which requires a high degree of responsiveness in healthcare systems, but also in workplaces, schools, and society. In this respect, the Headway Mental Health Index proved useful in assessing up to 54 MH-related KPIs across European countries, which may also monitor changes over time. This study’s findings may therefore guide future evidence-based European MH policies.

MH has become a top priority for European institutions [31]. It is therefore in our hands not to miss this unique opportunity to make a change in European MH policymaking. Enhancing our countries’ responsiveness to their citizens’ MH needs will benefit both the current and future generations.

## Supporting information

10.1192/j.eurpsy.2025.2448.sm001 Arango et al. supplementary material Arango et al. supplementary material

## Data Availability

All the data supporting the findings of this study are available in the online Supplementary Material.

## Supplementary material

The supplementary material for this article can be found at http://doi.org/10.1192/j.eurpsy.2025.2448.
