# Supplementary material for: Responsiveness of European countries to the population mental health needs: A cross-national comparison study
Source: Eur Psychiatry. 2025 Apr 14;68(1):e102. doi: 10.1192/j.eurpsy.2025.2448 (PMC12344467; doi:10.1192/j.eurpsy.2025.2448)
Supplement: Arango et al. supplementary material [file S0924933825024484sup001.zip › Table_S7_Respons_Society (1).doc]

| **Table S7. Responsiveness to mental health needs in society** | | | | | | | | | | | | | |
| --- | --- | --- | --- | --- | --- | --- | --- | --- | --- | --- | --- | --- | --- |
|  | **Social workers** | | **Occupational Therapists** | | **Social Support** | | **Hospital beds** | | **Disability benefits** | | **MH promotion programms** | | **Final score** |
|  | Raw | HI | Raw | HI | Raw | HI | Raw | HI | Raw | HI | Raw | HI | HI |
| Austria |  | 2,1 | 51,0 | 3,2 | 6,4 | 8,3 | 691,5 | 8,5 | 294,15 | 3,2 | 0,250 | 3,3 | 7,4 |
| Belgium | 0,89 | 1,4 | 124,2 | 6,5 | 14,9 | 5,0 | 547,2 | 6,3 | 405,68 | 4,2 | 0,500 | 5,5 | 6,1 |
| Bulgaria | 0,71 | 1,3 | 1,0 | 1,0 | 14,5 | 5,1 | 792,3 | 10,0 | 60,14 | 1,2 | 0,000 | 1,0 | 4,5 |
| Croatia | 1,33 | 1,6 | 16,9 | 1,7 | 12 | 6,1 | 567,5 | 6,6 | 104,09 | 1,6 | 0,000 | 1,0 | 3,7 |
| Cyprus | 0,00 | 1,0 | 32,7 | 2,4 | 2,4 | 9,9 | 312,4 | 2,7 | 67,52 | 1,3 | 0,100 | 1,9 | 3,7 |
| Czechia | 1,12 | 1,5 | 13,0 | 1,5 | 10,2 | 6,8 | 665,5 | 8,1 | 100,90 | 1,6 | 0,750 | 7,8 | 7,5 |
| Denmark |  | 2,1 | 204,3 | 10,0 | 18,7 | 3,5 | 251,3 | 1,8 | 1069,62 | 10,0 | 0,750 | 7,8 | 7,2 |
| Estonia | 0,53 | 1,3 | 11,3 | 1,5 | 23 | 1,8 | 439,4 | 4,6 | 159,13 | 2,1 | 0,250 | 3,3 | 2,0 |
| Finland | 2,75 | 2,3 | 66,7 | 3,9 | 22 | 2,1 | 282,6 | 2,3 | 461,86 | 4,7 | 0,750 | 7,8 | 4,7 |
| France | 10,76 | 6,3 | 20,9 | 1,9 | 12 | 6,1 | 564,8 | 6,5 | 276,35 | 3,1 | 0,750 | 7,8 | 9,3 |
| Germany |  | 2,1 | 73,3 | 4,2 | 13,9 | 5,4 | 776,0 | 9,8 | 412,55 | 4,3 | 1,000 | 10,0 | 10,0 |
| Greece | 3,46 | 2,7 | 16,5 | 1,7 | 24,2 | 1,3 | 426,7 | 4,4 | 65,48 | 1,2 | 0,000 | 1,0 | 1,0 |
| Hungary | 2,98 | 2,5 |  | 2,2 | 12,2 | 6,0 | 678,6 | 8,3 | 51,16 | 1,1 | 0,750 | 7,8 | 7,5 |
| Ireland | 7,09 | 4,5 | 66,4 | 3,9 | 5,1 | 8,9 | 289,4 | 2,4 | 208,23 | 2,5 | 0,500 | 5,5 | 6,6 |
| Italy | 2,41 | 2,2 | 4,5 | 1,2 | 15,8 | 4,6 | 312,4 | 2,7 | 181,22 | 2,3 | 0,500 | 5,5 | 3,9 |
| Latvia | 4,10 | 3,0 | 11,2 | 1,5 | 24,2 | 1,3 | 516,4 | 5,8 | 99,74 | 1,5 | 0,750 | 7,8 | 4,8 |
| Lithuania | 3,75 | 2,8 | 17,8 | 1,7 | 13 | 5,7 | 605,4 | 7,2 | 105,08 | 1,6 | 0,750 | 7,8 | 7,2 |
| Luxembourg |  | 2,1 | 58,9 | 3,6 | 15,6 | 4,7 | 414,3 | 4,3 | 1027,25 | 9,6 | 0,250 | 3,3 | 6,7 |
| Malta | 5,12 | 3,5 | 29,4 | 2,3 | 10,3 | 6,8 | 414,4 | 4,3 | 65,21 | 1,2 | 0,100 | 1,9 | 4,0 |
| Netherlands |  | 2,1 | 35,8 | 2,5 | 14,7 | 5,0 | 295,2 | 2,4 | 447,30 | 4,6 | 0,100 | 1,9 | 3,3 |
| Poland | 0,62 | 1,3 | 1,5 | 1,0 | 10,9 | 6,6 | 627,2 | 7,5 | 81,19 | 1,4 | 0,500 | 5,5 | 6,0 |
| Portugal | 0,84 | 1,4 | 25,2 | 2,1 | 13,4 | 5,6 | 351,1 | 3,3 | 133,84 | 1,8 | 1,000 | 10,0 | 6,0 |
| Romania | 0,50 | 1,2 | 1,1 | 1,0 | 24,9 | 1,0 | 720,6 | 8,9 | 37,59 | 1,0 | 0,250 | 3,3 | 3,1 |
| Slovakia |  | 2,1 |  | 2,2 | 19,9 | 3,0 | 567,5 | 6,6 | 100,17 | 1,5 | 0,250 | 3,3 | 3,5 |
| Slovenia | 1,92 | 1,9 | 29,9 | 2,3 | 12,4 | 6,0 | 424,9 | 4,4 | 101,92 | 1,6 | 0,750 | 7,8 | 5,7 |
| Spain | 0,11 | 1,1 | 22,2 | 1,9 | 2,2 | 10,0 | 296,1 | 2,5 | 159,46 | 2,1 | 0,250 | 3,3 | 4,5 |
| Sweden | 18,42 | 10,0 | 118,0 | 6,2 | 15 | 4,9 | 200,1 | 1,0 | 450,15 | 4,6 | 0,750 | 7,8 | 8,6 |
| UK | 4,75 | 3,3 | 61,8 | 3,7 | 9,2 | 7,2 | 250,00 | 1,8 | 218,82 | 2,6 | 0,750 | 7,8 | 6,2 |
| EU-27+UK | 3,13 |  | 38,32 |  | 12,51 |  | 487,37 |  | 246,18 |  | 0,61 |  |  |
