# Supplementary material for: Responsiveness of European countries to the population mental health needs: A cross-national comparison study
Source: Eur Psychiatry. 2025 Apr 14;68(1):e102. doi: 10.1192/j.eurpsy.2025.2448 (PMC12344467; doi:10.1192/j.eurpsy.2025.2448)
Supplement: Arango et al. supplementary material [file S0924933825024484sup001.zip › Table_S1_HC_Workforce (1).doc]

| **Table S1. KPIs in responsiveness to MH needs in healthcare workforce: raw data and Headway scores** | | | | | | | | | |
| --- | --- | --- | --- | --- | --- | --- | --- | --- | --- |
|  | **Psychiatrists** | | **Child psychiatrists** | | **Psychologists** | | **Nurses** | | **Final score** |
|  | Raw | HI | Raw | HI | Raw | HI | Raw | HI | HI |
| Austria | 22,09 | 6,8 | *1,08* | 2,4 | 78,88 | 6,7 | 41,00 | 3,3 | 5,6 |
| Belgium | 17,36 | 4,5 | *2,22* | 3,8 | 10,46 | 1,7 | 125,69 | 8,4 | 5,4 |
| Bulgaria | 10,19 | 1,0 | 0,31 | 1,4 | 1,27 | 1,0 | 17,74 | 1,9 | 1,0 |
| Croatia | 17,34 | 4,5 | 1,62 | 3,0 | 5,67 | 1,3 | 41,84 | 3,3 | 3,3 |
| Cyprus | 13,55 | 2,6 | 1,71 | 3,1 | 22,80 | 2,6 | 34,97 | 2,9 | 3,0 |
| Czechia | 16,61 | 4,1 | 0,85 | 2,1 | 3,35 | 1,2 | 30,35 | 2,6 | 2,6 |
| Denmark | 19,19 | 5,4 | 4,75 | 6,9 | 54,00 | 4,9 | 153,00 | 10,0 | 8,3 |
| Estonia | 19,23 | 5,4 | 2,26 | 3,8 | 13,89 | 1,9 | 23,48 | 2,2 | 3,7 |
| Finland | 25,05 | 8,3 | *7,20* | 10,0 | 123,62 | 10,0 | 51,97 | 3,9 | 10,0 |
| France | 22,74 | 7,1 | 2,84 | 4,6 | 11,72 | 1,8 | 98,02 | 6,7 | 6,0 |
| Germany | 28,56 | 10,0 | 2,76 | 4,5 | 55,08 | 5,0 | 56,10 | 4,2 | 7,1 |
| Greece | 24,94 | 8,2 | 1,36 | 2,7 | 8,78 | 1,6 | 12,75 | 1,6 | 3,9 |
| Hungary | 14,36 | 3,0 | 0,53 | 1,7 | 15,85 | 2,1 | 33,74 | 2,9 | 2,5 |
| Ireland | 19,07 | 5,4 | 2,81 | 4,5 | 7,95 | 1,5 | 99,09 | 6,8 | 5,3 |
| Italy | 20,36 | 6,0 | *3,40* | 5,3 | 3,94 | 1,2 | 23,49 | 2,2 | 4,1 |
| Latvia | 14,81 | 3,3 | 0,65 | 1,8 | 31,52 | 3,2 | 22,83 | 2,2 | 2,7 |
| Lithuania | 24,67 | 8,1 | 3,17 | 5,0 | 18,95 | 2,3 | 49,76 | 3,8 | 5,6 |
| Luxembourg | 21 | 6,3 | 3,18 | 5,0 | 48,90 | 4,5 | 35,00 | 2,9 | 5,5 |
| Malta | 13,89 | 2,8 | 0,00 | 1,0 | 4,39 | 1,2 | 66,83 | 4,8 | 2,5 |
| Netherlands | 25,06 | 8,3 | 2,60 | 4,3 | 123,46 | 10,0 | 132,30 | 8,8 | 9,7 |
| Poland | 13,02 | 2,4 | 1,57 | 3,0 | 12,19 | 1,8 | 30,92 | 2,7 | 2,5 |
| Portugal | 14,64 | 3,2 | *0,97* | 2,2 | 2,56 | 1,1 | 10,87 | 1,5 | 1,9 |
| Romania | 15,27 | 3,5 | 0,56 | 1,7 | 1,48 | 1,0 | 18,71 | 1,9 | 2,0 |
| Slovakia | 11 | 1,4 | 0,82 | 2,0 | 18,32 | 2,3 | 36,73 | 3,0 | 2,1 |
| Slovenia | 16,37 | 4,0 | 0,72 | 1,9 | 12,08 | 1,8 | 36,73 | 3,0 | 2,8 |
| Spain | 12,58 | 2,2 | 0,00 | 1,0 | 63,90 | 5,6 | 2,87 | 1,0 | 2,5 |
| Sweden | 21,98 | 6,8 | 3,42 | 5,3 | 84,88 | 7,2 | 50,57 | 3,9 | 6,9 |
| UK | 20 | 5,8 | 1,00 | 2,3 | 19,77 | 2,4 | 55,62 | 4,2 | 4,1 |
| EU-27+UK | 20,20 |  | 2,00 |  | 31,39 |  | 51,14 |  |  |

HI: Headway-transformed scores
