# Supplementary material for: Responsiveness of European countries to the population mental health needs: A cross-national comparison study
Source: Eur Psychiatry. 2025 Apr 14;68(1):e102. doi: 10.1192/j.eurpsy.2025.2448 (PMC12344467; doi:10.1192/j.eurpsy.2025.2448)
Supplement: Arango et al. supplementary material [file S0924933825024484sup001.zip › Table_S2_H_Facilities (1).doc]

| **Table S2. KPIs in Responsiveness to MH needs in healthcare facilities: raw data and Headway-transformed scores** | | | | | | | | | | | |
| --- | --- | --- | --- | --- | --- | --- | --- | --- | --- | --- | --- |
|  | **Psychiatric hospital beds** | | **Child psychiatric beds** | | **Mental hospitals** | | **Mental health units in general hospitals** | | **Mental health outpatient facilities** | | **Final score** |
|  | Raw | HI | Raw | HI | Raw | HI | Raw | HI | Raw | HI | HI |
| Austria | 71,8 | 5,7 | 19,3 | 7,1 | 0,092 | 8,7 | 0,265 | 9,0 | 3,457 | 1,6 | 7,3 |
| Belgium | 141,5 | 1,0 | 26,5 | 6,0 | 0,442 | 3,9 | 0,077 | 9,9 | 0,693 | 1,1 | 3,4 |
| Bulgaria | 57,2 | 6,7 | 3,8 | 9,6 | 0,171 | 7,6 | 0,314 | 8,7 | 0,986 | 1,2 | 7,9 |
| Croatia | 94,0 | 4,2 | 9,9 | 8,6 | 0,194 | 7,3 | 0,533 |  | 33,363 | 6,9 | 8,0 |
| Cyprus | 18,4 | 9,3 | 4,4 | 9,5 | 0,086 | 8,8 | 0,172 | 9,4 | 0,861 | 1,1 | 9,6 |
| Czechia | 85,6 | 4,8 | 29,9 | 5,4 | 0,215 | 7,0 | 0,299 | 8,8 | 11,582 | 3,0 | 6,1 |
| Denmark | 51,9 | 7,0 | 18,4 | 7,3 | 0,100 | 8,6 |  |  |  | 1,6 | 6,7 |
| Estonia | 50,2 | 7,2 | 18,4 | 7,3 | 0,152 | 7,9 | 0,684 | 7,0 | 14,820 | 3,6 | 7,6 |
| Finland | 37,8 | 8,0 | 31,9 | 5,1 | 0,054 | 9,3 | 0,560 | 7,6 | 6,977 | 2,2 | 7,3 |
| France | 79,2 | 5,2 | 13,8 | 8,0 | 0,588 | 1,9 | 0,359 | 8,5 | 10,229 | 2,8 | 5,1 |
| Germany | 130,9 | 1,7 | 57,4 | 1,0 | 0,335 | 5,4 | 0,491 | 7,9 | 0,077 | 1,0 | 1,5 |
| Greece | 70,0 | 5,8 | 3,2 | 9,7 | 0,029 | 9,6 | 0,391 | 8,4 | 2,702 | 1,5 | 8,4 |
| Hungary | 77,4 | 5,3 | 7,7 | 9,0 | 0,010 | 9,9 | 1,931 | 1,0 | 12,928 | 3,3 | 5,9 |
| Ireland | 32,1 | 8,4 | 4,8 | 9,5 | *0,655* | 1,0 | 0,451 | 8,1 | 3,728 | 1,6 | 5,9 |
| Italy | 8,0 | 10,0 | 3,3 | 9,7 | 0,000 | 10,0 | 0,563 | 7,5 | 3,410 | 1,6 | 9,9 |
| Latvia | 110,7 | 3,1 | 37,3 | 4,2 | 0,472 | 3,5 | 0,151 | 9,5 | 14,475 | 3,6 | 4,1 |
| Lithuania | 87,3 | 4,7 | 34,5 | 4,7 | 0,217 | 7,0 | 0,688 | 6,9 | 50,804 | 10,0 | 7,7 |
| Luxembourg | 79,8 | 5,2 | 27,1 | 5,9 | 0,176 | 7,6 | 0,706 | 6,9 | 3,176 | 1,5 | 5,3 |
| Malta | 71,9 | 5,7 | 13,8 | 8,0 | 0,490 | 3,3 |  |  | 0,490 | 1,1 | 3,6 |
| Netherlands | 112,3 | 3,0 | 56,4 | 1,2 | 0,201 | 7,2 |  |  | 1,190 | 1,2 | 1,0 |
| Poland | 60,7 | 6,4 | 17,9 | 7,3 | 0,121 | 8,3 | 0,404 | 8,3 | 6,625 | 2,2 | 7,5 |
| Portugal | 64,0 | 6,2 | 1,4 | 10,0 | 0,020 | 9,7 | 0,323 | 8,7 | 1,125 | 1,2 | 8,7 |
| Romania | 86,3 | 4,7 | 17,5 | 7,4 | 0,171 | 7,7 | 0,392 | 8,4 | 0,911 | 1,1 | 6,2 |
| Slovakia | 79,7 | 5,2 | 20,3 | 7,0 | 0,170 | 7,7 |  |  | 6,390 | 2,1 | 5,5 |
| Slovenia | 64,0 | 6,2 | 11,7 | 8,3 | 0,241 | 6,7 | 0,048 | 10,0 | 39,016 | 7,9 | 10,0 |
| Spain | 36,0 | 8,1 | 2,4 | 9,8 | 0,183 | 7,5 | 1,028 | 5,3 | 2,045 | 1,3 | 7,3 |
| Sweden | 39,8 | 7,9 | 6,8 | 9,1 | 0,000 | 10,0 |  |  |  | 1,6 | 8,7 |
| UK | 35,00 | 8,2 | 8,5 | 8,9 | 0,201 | 7,2 |  |  | 4,940 | 1,9 | 7,5 |
| EU-27+UK | 68,19 |  | 20,03 |  | 0,23 |  | 0,42 |  | 4,77 |  |  |

HI: Headway-transformed scores
