# Supplementary material for: Responsiveness of European countries to the population mental health needs: A cross-national comparison study
Source: Eur Psychiatry. 2025 Apr 14;68(1):e102. doi: 10.1192/j.eurpsy.2025.2448 (PMC12344467; doi:10.1192/j.eurpsy.2025.2448)
Supplement: Arango et al. supplementary material [file S0924933825024484sup001.zip › Table_S3_HC_QoC (1).doc]

| **Table S3. Responsiveness to mental health needs in healthcare quality of care** | | | | | | | | | | | |
| --- | --- | --- | --- | --- | --- | --- | --- | --- | --- | --- | --- |
|  | **Hospital discharge rates** | | **Length of stay** | | **MH consultations** | | **Unmet needs** | | **Psychiatric articles** | | **Final score** |
|  | Raw | HI | Raw | HI | Raw | HI | Raw | HI | Raw | HI | HI |
| Austria | 1.144,1 | 3,9 | 26,70 | 7,8 | 7,40 | 6,7 | 6,20 | 8,4 | 5,72 | 4,6 | 5,1 |
| Belgium | 303,5 | 8,6 | 9,10 | 10,0 | 9,50 | 8,5 | 6,17 | 8,4 | 6,00 | 4,8 | 8,2 |
| Bulgaria | 550,1 | 7,2 | 43,40 | 5,6 | 1,50 | 1,5 | 5,10 | 8,7 | 1,29 | 1,5 | 2,8 |
| Croatia | 928,1 | 5,1 | 17,70 | 8,9 | 10,40 | 9,3 | 2,90 | 9,4 | 3,52 | 3,0 | 6,6 |
| Cyprus | 82,3 | 9,8 | 26,60 | 7,8 | 9,40 | 8,4 | 4,70 | 8,9 | 9,94 | 7,5 | 8,9 |
| Czechia | 657,7 | 6,6 | 15,70 | 9,2 | 3,90 | 3,6 | 2,40 | 9,6 | 2,45 | 2,3 | 5,1 |
| Denmark | 889,0 | 5,3 | 20,50 | 8,6 | 10,40 | 9,3 | 15,50 | 5,5 | 13,52 | 10,0 | 7,6 |
| Estonia | 939,0 | 5,0 | 24,20 | 8,1 | 8,10 | 7,3 | 3,30 | 9,3 | 3,83 | 3,3 | 5,7 |
| Finland | 1.205,2 | 3,5 | 26,90 | 7,7 | 9,20 | 8,3 | 29,80 | 1,0 | 7,79 | 6,0 | 3,5 |
| France | 1.149,0 | 3,8 | 80,00 | 1,0 | 7,20 | 6,5 | 7,30 | 8,1 | 2,96 | 2,6 | 1,9 |
| Germany | 1.653,3 | 1,0 | 31,30 | 7,2 | 10,90 | 9,7 | 3,80 | 9,2 | 4,66 | 3,8 | 5,0 |
| Greece | 321,4 | 8,5 | 11,60 | 9,7 | 4,10 | 3,8 | 4,60 | 8,9 | 2,98 | 2,7 | 5,9 |
| Hungary | 873,8 | 5,4 | 14,10 | 9,4 | 4,70 | 4,3 | 2,60 | 9,5 | 2,71 | 2,5 | 5,0 |
| Ireland | 149,8 | 9,4 | 24,90 | 8,0 | 4,70 | 4,3 | 5,20 | 8,7 | 12,67 | 9,4 | 8,0 |
| Italy | 272,3 | 8,7 | 19,60 | 8,7 | 3,50 | 3,3 | 2,60 | 9,5 | 4,38 | 3,6 | 6,0 |
| Latvia | 1.202,6 | 3,5 | 26,80 | 7,8 | 4,30 | 4,0 | 5,50 | 8,6 | 1,28 | 1,5 | 3,1 |
| Lithuania | 1.247,3 | 3,3 | 9,50 | 9,9 | 6,00 | 5,5 | 2,80 | 9,5 | 2,64 | 2,4 | 4,9 |
| Luxembourg | 1.099,0 | 4,1 | 36,60 | 6,5 | 9,90 | 8,9 | 4,80 | 8,8 | 5,42 | 4,4 | 5,6 |
| Malta | 478,5 | 7,6 | 26,60 | 7,8 | 5,30 | 4,8 | 1,60 | 9,8 | 3,84 | 3,3 | 5,8 |
| Netherlands | 45,9 | 10,0 | 20,00 | 8,6 | 9,80 | 8,8 | 2,20 | 9,7 | 11,48 | 8,6 | 10,0 |
| Poland | 695,3 | 6,4 | 28,50 | 7,5 | 4,10 | 3,8 | 3,00 | 9,4 | 1,55 | 1,7 | 4,2 |
| Portugal | 182,8 | 9,2 | 23,40 | 8,2 | 7,30 | 6,6 | 27,80 | 1,6 | 4,93 | 4,0 | 4,5 |
| Romania | 1.317,1 | 2,9 | 53,10 | 4,4 | 0,90 | 1,0 | 1,10 | 10,0 | 0,60 | 1,0 | 1,0 |
| Slovakia | 816,5 | 5,7 | 15,60 | 9,2 | 3,90 | 3,6 | 2,10 | 9,7 | 1,16 | 1,4 | 4,5 |
| Slovenia | 572,2 | 7,1 | 24,80 | 8,0 | 5,80 | 5,3 | 3,40 | 9,3 | 2,66 | 2,4 | 5,4 |
| Spain | 250,3 | 8,9 | 35,20 | 6,7 | 4,80 | 4,4 | 1,20 | 10,0 | 3,83 | 3,2 | 5,7 |
| Sweden | 962,8 | 4,9 | 28,00 | 7,6 | 11,20 | 10,0 | 9,00 | 7,5 | 11,60 | 8,7 | 7,6 |
| UK | 285,7 | 8,7 | 24 | 8,2 | 3,6 | 3,4 | 6,17 | 8,4 | 9,37 | 7,1 | 6,6 |
| EU27+UK | 762,66 |  | 33,62 |  | 6,21 |  | 5,06 |  | 5,00 |  |  |

HI: Headway Initiative scores
