# Supplementary material for: Responsiveness of European countries to the population mental health needs: A cross-national comparison study
Source: Eur Psychiatry. 2025 Apr 14;68(1):e102. doi: 10.1192/j.eurpsy.2025.2448 (PMC12344467; doi:10.1192/j.eurpsy.2025.2448)
Supplement: Arango et al. supplementary material [file S0924933825024484sup001.zip › Table_S4_HC_Expenditure (1) (1).doc]

| **Table S4. Responsiveness to mental health needs in healthcare economic resources** | | |
| --- | --- | --- |
|  | **% of total health expenditure** | |
|  | Raw | HI |
| Austria | 4,3 | 2,7 |
| Belgium | 3,5 | 2,1 |
| Bulgaria | 2,0 | 1,0 |
| Croatia | 4,3 | 2,7 |
| Cyprus | 4,8 | 3,1 |
| Czechia | 4,1 | 2,6 |
| Denmark | 5,4 | 3,6 |
| Estonia | 2,9 | 1,7 |
| Finland | 6,3 | 4,3 |
| France | 13,9 | 10,0 |
| Germany | 13,1 | 9,4 |
| Greece | 3,0 | 1,8 |
| Hungary | 3,2 | 1,9 |
| Ireland | 5,1 | 3,3 |
| Italy | 3,0 | 1,7 |
| Latvia | 4,8 | 3,1 |
| Lithuania | 4,2 | 2,7 |
| Luxembourg | 8,3 | 5,8 |
| Malta | 6,7 | 4,6 |
| Netherlands | 4,5 | 2,9 |
| Poland | 3,1 | 1,8 |
| Portugal | 5,2 | 3,5 |
| Romania | 3,0 | 1,8 |
| Slovakia | 3,6 | 2,2 |
| Slovenia | 5,8 | 3,9 |
| Spain | 5,0 | 3,3 |
| Sweden | 10,0 | 7,1 |
| UK | 7,6 | 5,2 |
| EU-27+UK | 8,7 |  |
