# Supplementary material for: Responsiveness of European countries to the population mental health needs: A cross-national comparison study
Source: Eur Psychiatry. 2025 Apr 14;68(1):e102. doi: 10.1192/j.eurpsy.2025.2448 (PMC12344467; doi:10.1192/j.eurpsy.2025.2448)
Supplement: Arango et al. supplementary material [file S0924933825024484sup001.zip › Table_S5_Raw_Data_Workplaces (1).doc]

| **Table S5. Responsiveness to mental health needs in workplaces** | | | | | | | | | | | | |  |
| --- | --- | --- | --- | --- | --- | --- | --- | --- | --- | --- | --- | --- | --- |
|  | **Gross wage** | | **Employment rate** | | **Sick leave benefits** | | **Unemployment benefits** | | **Job quality** | | **MH programas promotion** | | **Final score** |
|  | Raw | HI | Raw | HI | Raw | HI | Raw | HI | Raw | HI | Raw | HI | HI |
| Austria | 84,6 | 7,4 | 39,4 | 4,2 | 97,94 | 4,4 | 200,26 | 10,0 | 7,4 | 7,3 | 0,000 | 1,0 | 7,8 |
| Belgium | 89,0 | 9,3 | 44,7 | 5,8 | 73,28 | 3,5 | 166,35 | 8,5 | 7,0 | 6,7 | 0,500 | 5,5 | 9,4 |
| Bulgaria |  | 6,5 |  | 5,2 | 8,85 | 1,1 | 7,82 | 1,2 | 6,8 | 6,4 | 0,000 | 1,0 | 3,7 |
| Croatia |  | 6,5 |  | 5,2 | 28,48 | 1,9 | 18,46 | 1,7 | 4,5 | 2,7 | 0,850 | 8,7 | 5,4 |
| Cyprus |  | 6,5 |  | 5,2 | 22,21 | 1,6 | 137,67 | 7,1 | 5,9 | 4,9 | 0,100 | 1,9 | 5,6 |
| Czechia |  | 6,5 |  | 5,2 | 27,62 | 1,8 | 36,83 | 2,6 | 4,9 | 3,4 | 0,000 | 1,0 | 3,4 |
| Denmark | 81,2 | 5,9 | 41,3 | 4,8 | 76,42 | 3,6 | 101,67 | 5,5 | 7,6 | 7,7 | 1,000 | 10,0 | 8,9 |
| Estonia | 79,3 | 5,1 | 53,6 | 8,6 | 13,97 | 1,3 | 56,68 | 3,5 | 6,3 | 5,6 | 0,000 | 1,0 | 4,9 |
| Finland | 87,9 | 8,8 | 37,1 | 3,4 | 77,73 | 3,6 | 144,00 | 7,4 | 7,2 | 7,0 | 0,850 | 8,7 | 9,4 |
| France | 87,0 | 8,4 | 45,8 | 6,2 | 53,47 | 2,8 | 164,71 | 8,4 | 4,7 | 3,1 | 0,850 | 8,7 | 8,9 |
| Germany |  | 6,5 | 58,2 | 10,0 | 175,12 | 7,1 | 104,45 | 5,6 | 6,7 | 6,2 | 0,500 | 5,5 | 10,0 |
| Greece | 80,1 | 5,4 | 29,1 | 1,0 | 7,47 | 1,1 | 32,27 | 2,3 | 4,1 | 2,1 | 0,000 | 1,0 | 1,0 |
| Hungary | 82,4 | 6,4 | 32,8 | 2,1 | 12,98 | 1,3 | 9,34 | 1,3 | 5,1 | 3,6 | 0,500 | 5,5 | 3,4 |
| Ireland | 76,8 | 4,0 |  | 5,2 | 40,79 | 2,3 | 181,91 | 9,2 | 7,2 | 6,9 | 0,750 | 7,8 | 8,2 |
| Italy | 82,6 | 6,6 | 40,2 | 4,4 | 24,20 | 1,7 | 126,50 | 6,6 | 5,5 | 4,4 | 0,500 | 5,5 | 6,2 |
| Latvia | 84,1 | 7,2 | 47,8 | 6,8 | 20,87 | 1,6 | 28,00 | 2,2 | 6,1 | 5,2 | 0,000 | 1,0 | 4,5 |
| Lithuania | 76,8 | 4,0 | 37,4 | 3,6 | 26,38 | 1,8 | 54,97 | 3,4 | 4,9 | 3,3 | 0,850 | 8,7 | 4,8 |
| Luxembourg | 81,6 | 6,1 | 52,8 | 8,3 | 254,51 | 10,0 | 147,44 | 7,6 | 6,2 | 5,4 | 0,000 | 1,0 | 9,2 |
| Malta |  | 6,5 |  | 5,2 | 36,52 | 2,1 | 105,73 | 5,7 | 7,2 | 7,0 | 0,100 | 1,9 | 6,0 |
| Netherlands | 90,5 | 10,0 | 45,4 | 6,0 | 172,41 | 7,0 | 55,64 | 3,4 | 9,1 | 10,0 | 0,100 | 1,9 | 9,2 |
| Poland | 90,6 | 10,0 | 44,8 | 5,8 | 24,03 | 1,7 | 15,44 | 1,6 | 4,4 | 2,5 | 0,850 | 8,7 | 6,6 |
| Portugal | 69,8 | 1,0 | 44,3 | 5,7 | 17,36 | 1,5 | 46,04 | 3,0 | 6,4 | 5,8 | 0,850 | 8,7 | 5,0 |
| Romania |  | 6,5 |  | 5,2 | 4,85 | 1,0 | 2,67 | 1,0 | 7,5 | 7,4 | 0,000 | 1,0 | 4,0 |
| Slovakia |  | 6,5 |  | 5,2 | 22,17 | 1,6 | 21,67 | 1,9 | 3,4 | 1,0 | 0,000 | 1,0 | 2,4 |
| Slovenia | 84,7 | 7,5 | 40,7 | 4,6 | 51,31 | 2,7 | 54,55 | 3,4 | 5,5 | 4,3 | 0,000 | 1,0 | 4,3 |
| Spain | 84,9 | 7,6 | 31,9 | 1,9 | 56,85 | 2,9 | 126,01 | 6,6 | 4,3 | 2,4 | 0,250 | 3,3 | 4,7 |
| Sweden | 79,6 | 5,2 | 50,8 | 7,7 | 117,63 | 5,1 | 73,07 | 4,2 | 6,8 | 6,4 | 0,000 | 1,0 | 6,4 |
| UK | 76,5 | 3,9 | 35,4 | 2,9 | 37,85 | 2,2 | 16,87 | 1,6 | 5,7 | 4,7 | 0,850 | 8,7 | 4,5 |
| EU-27+UK | 62,3 |  | 38,9 |  | 66,9 |  | 87,9 |  | 5,7 |  | 2,9 |  |  |
