# Supplementary material for: Responsiveness of European countries to the population mental health needs: A cross-national comparison study
Source: Eur Psychiatry. 2025 Apr 14;68(1):e102. doi: 10.1192/j.eurpsy.2025.2448 (PMC12344467; doi:10.1192/j.eurpsy.2025.2448)
Supplement: Arango et al. supplementary material [file S0924933825024484sup001.zip › Table_S6_Responsiveness_Schools (1).doc]

| **Table S6. KPIs in Responsiveness to mental health needs in schools** | | | | | | | |
| --- | --- | --- | --- | --- | --- | --- | --- |
|  | **Community facilities** | | **Drop-outs** | | **MH Promotion programms** | | **Final score** |
|  | Raw | HI | Raw | HI | Raw | HI | HI |
| Austria | 0,303 | 1,5 | 11,5% | 7,4 | 1,000 | 10,0 | 7,6 |
| Belgium | 0,174 | 1,2 | 18,2% | 3,1 | 0,600 | 6,4 | 4,0 |
| Bulgaria | 0,101 | 1,1 | 11,1% | 7,7 | 0,400 | 4,6 | 5,2 |
| Croatia | 0,591 | 1,9 | 9,7% | 8,6 | 0,600 | 6,4 | 6,7 |
| Cyprus | 0,450 | 1,7 | 18,1% | 3,2 | 0,100 | 1,9 | 2,2 |
| Czechia |  | 2,0 | 18,8% | 2,7 | 0,600 | 6,4 | 4,1 |
| Denmark | 2,000 | 4,2 | 16,0% | 4,5 | 1,000 | 10,0 | 7,5 |
| Estonia |  | 2,0 | 7,5% | 10,0 | 0,600 | 6,4 | 7,4 |
| Finland | 0,652 | 2,0 | 12,6% | 6,7 | 1,000 | 10,0 | 7,5 |
| France | 3,801 | 7,1 | 11,8% | 7,3 | 1,000 | 10,0 | 10,0 |
| Germany | 0,402 | 1,6 | 9,9% | 8,5 | 1,000 | 10,0 | 8,1 |
| Greece | 0,504 | 1,8 | 16,1% | 4,5 | 0,000 | 1,0 | 2,4 |
| Hungary | 0,993 | 2,6 | 8,3% | 9,5 | 1,000 | 10,0 | 9,0 |
| Ireland | 1,430 | 3,3 | 18,8% | 2,7 | 1,000 | 10,0 | 6,3 |
| Italy | 1,174 | 2,9 | 10,7% | 8,0 | 0,600 | 6,4 | 6,8 |
| Latvia |  | 2,0 | 8,7% | 9,2 | 1,000 | 10,0 | 8,6 |
| Lithuania |  | 2,0 | 8,7% | 9,3 | 1,000 | 10,0 | 8,6 |
| Luxembourg | 0,799 | 2,3 | 12,4% | 6,9 | 0,000 | 1,0 | 3,7 |
| Malta | 0,480 | 1,7 | 11,3% | 7,6 | 0,100 | 1,9 | 4,2 |
| Netherlands | 5,630 | 10,0 | 21,5% | 1,0 | 0,100 | 1,9 | 4,9 |
| Poland | 0,896 | 2,4 | 18,2% | 3,1 | 0,800 | 8,2 | 5,3 |
| Portugal | 0,398 | 1,6 | 12,8% | 6,6 | 1,000 | 10,0 | 7,3 |
| Romania | 0,124 | 1,2 | 11,1% | 7,7 | 0,600 | 6,4 | 6,0 |
| Slovakia | 0,018 | 1,0 | 19,9% | 2,0 | 0,000 | 1,0 | 1,0 |
| Slovenia | 1,162 | 2,8 | 9,7% | 8,6 | 0,800 | 8,2 | 7,9 |
| Spain | 0,990 | 2,6 | 16,5% | 4,2 | 0,600 | 6,4 | 5,1 |
| Sweden | 0,210 | 1,3 | 11,7% | 7,3 | 0,400 | 4,6 | 5,1 |
| UK | 1,480 | 3,3 | 11,7% | 7,3 | 1,000 | 10,0 | 8,4 |
| EU-27+UK | 1,36 |  | 13,0% |  | 0,78 |  |  |
